# Integrated analysis of hepatic mRNA and miRNA profiles identified molecular networks and potential biomarkers of NAFLD

**DOI:** 10.1038/s41598-018-25743-8

**Published:** 2018-05-16

**Authors:** Mingzhe Zhu, Qianlei Wang, Wenjun Zhou, Tao Liu, Lili Yang, Peiyong Zheng, Li Zhang, Guang Ji

**Affiliations:** 1 Institute of Digestive Diseases, Longhua Hospital, China-Canada Center of Research for Digestive Diseases (ccCRDD), Shanghai University of Traditional Chinese Medicine, Shanghai, 200032 China; 2 School of Public Health, Shanghai University of Traditional Chinese Medicine, Shanghai, 201203 China

## Abstract

To enhance our understanding of molecular mechanisms and mine novel biomarkers of non-alcoholic fatty liver disease (NAFLD), RNA sequencing was performed to gain hepatic expression profiles of mRNAs and miRNAs in NAFLD and normal rats. Using DESeq with thresholds of a two-fold change and a false discovery rate (FDR) less than 0.05, 336 mRNAs and 21 miRNAs were identified as differentially expressed. Among those, 17 miRNAs (e.g., miR-144-3p, miR-99a-3p, miR-200b-3p, miR-200b-5p, miR-200c-3p, *etc*.) might serve as novel biomarkers of NAFLD. MiRNA target genes (13565) were predicted by the miRWalk database. Using DAVID 6.8, the intersection (195 genes) of differentially expressed mRNAs and miRNA-predicted target genes were enriched in 47 gene ontology (GO) terms and 28 Kyoto Encyclopedia of Genes and Genomes (KEGG) pathways. Using Cytoscape, pathway interaction and protein-protein interaction (PPI) networks were constructed, and hub genes (e.g., Abcg8, Cyp1a1, Cyp51, Hmgcr, *etc*.) associated with NAFLD were obtained. Moreover, 673 miRNA-mRNA negative regulatory pairs were obtained, and networks were constructed. Finally, several representative miRNAs and mRNAs were validated by real-time qPCR. In conclusion, potential molecular mechanisms of NAFLD could be inferred from integrated analysis of mRNA and miRNA profiles, which may indicate novel biomarkers of NAFLD.

## Introduction

Nonalcoholic fatty liver disease (NAFLD) is the most common hepatic disease worldwide and is defined as evidence of hepatic steatosis, either by imaging or histology, with no cause for secondary hepatic fat accumulation such as significant alcohol consumption, use of steatogenic medication, or hereditary disorder^1^. The prevalence of NAFLD is 20–30% in Western countries, creating considerable concern with its progression from steatosis to steatohepatitis, cirrhosis, and even hepatocellular carcinoma^2^. Regarding the pathogenesis, the “multiple hits” hypothesis considers multiple insults in genetically predisposed subjects to induce NAFLD and provides a more accurate explanation of NAFLD pathogenesis than do other hypotheses. Such hits include insulin resistance, hormones secreted from adipose tissue, nutritional factors, gut microbiota and genetic and epigenetic factors, among others^3^. However, the molecular mechanisms in the pathogenesis of NAFLD are complex and still require further clarification.

Over the past decades, mRNA expression profiling has been used as a powerful tool to uncover molecular mechanisms and explore diagnostic and predictive biomarkers, particularly in complicated diseases such as cancer^4^, diabetes^5^, cardiovascular disease^6^ and NAFLD. One study reported that the expression of certain metabolism-related genes was induced and specific metabolic and repair pathways were activated in NAFLD^7^. Others reported that uptake transporter genes were coordinately targeted for down-regulation at the global level during the pathological development of NAFLD^8^. A recent study used high-throughput RNA sequencing to explore mRNA expression profiling by Western-diet-induced NAFLD and found that numerous lipid metabolism and cholesterol biosynthesis genes were altered^9^. Although numerous genes have been identified in NAFLD, the establishment of gene regulatory networks is limited.

MiRNAs are endogenous, single-stranded RNAs and have been widely known to regulate gene expression primarily through post-transcriptional mechanisms^10^. MiRNAs have multiple targets and regulate the expression of hundreds of genes that have important cellular functions such as cell differentiation, development, and proliferation and disease progression^11^. Alterations in miRNA expression profiles have been described in the pathophysiology of complex diseases^12–14^, and specific miRNA expression profiles have been shown to be associated with NAFLD. Several miRNAs, such as miR-122, miR-451, miR-200b and miR-27, have been found to be deregulated in different diet-induced NAFLD rat models^15^. Researchers have observed altered expression of miR-33a/miR-33b^*^ and miR-122 in liver samples, which have been designated possible contributors to hepatic lipid metabolism in patients with NAFLD^16^. However, studies on the identification of NAFLD-relevant miRNAs by high-throughput RNA sequencing are rare. By contrast, integrated analysis of mRNAs and miRNAs expression profiles in exploring miRNA-mRNA regulatory networks provides a powerful tool and facilitates clarification of the molecular mechanisms and identification of novel biomarkers of NAFLD.

In our previous study, we constructed NAFLD and normal rat models and observed different phenotypic characteristics^17^. To enhance our understanding of the mechanisms associated with the pathogenesis of NAFLD and mine potential biomarkers, here, we detected expression profiles of mRNAs and miRNAs in liver tissues and constructed pathway interaction, PPI and miRNA-mRNA regulatory networks through integrated analysis of the crucial mRNAs and miRNAs obtained. Our present study may pave a new way for understanding molecular mechanisms of NAFLD and uncover novel biomarkers.

## Results

### Quality control of RNA sequence experiment

**Figure 1:**
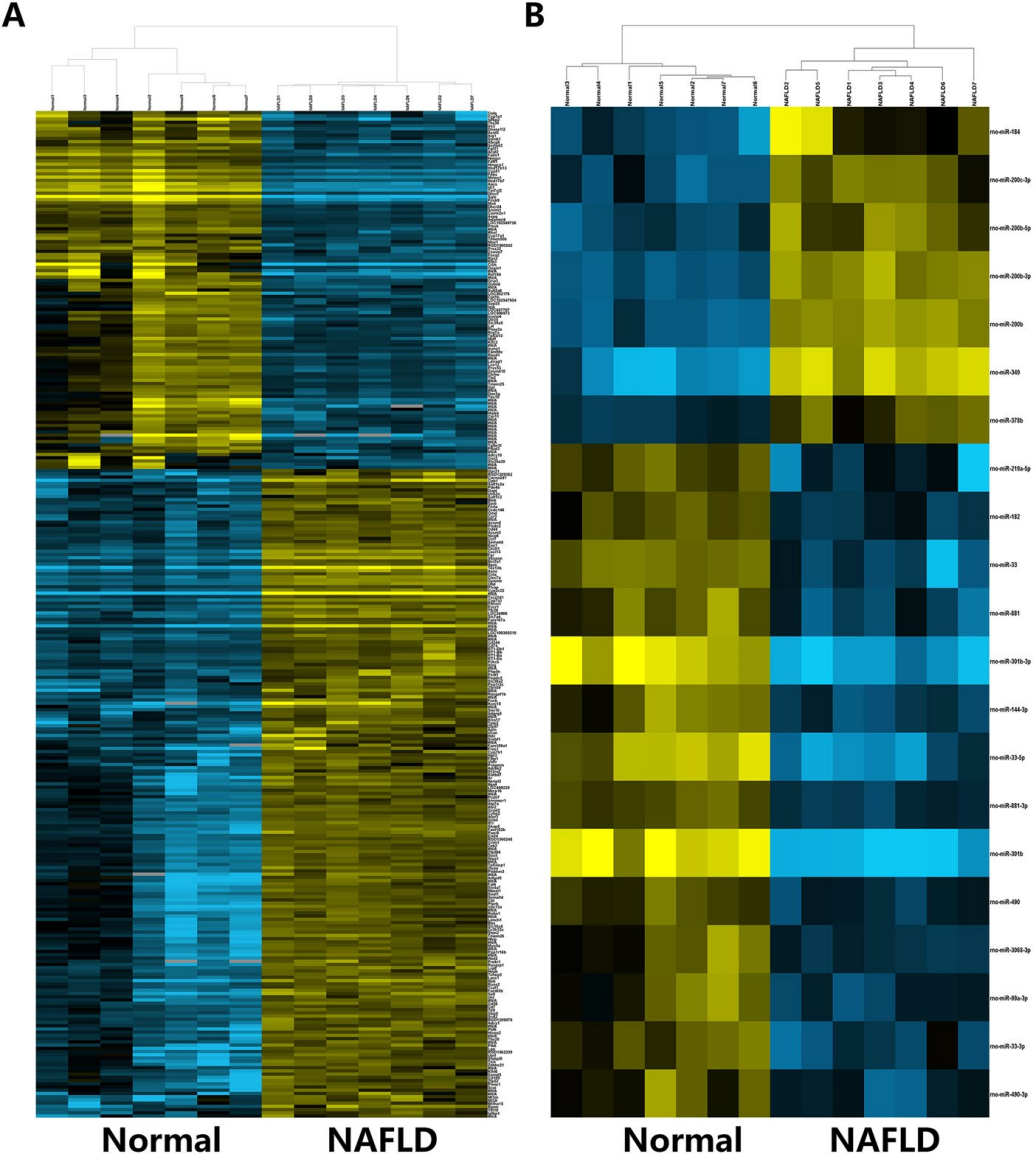
Hierarchical cluster of representative mRNA and miRNA expression across biological replicate samples. (**A**) Heatmap of representative mRNAs. (**B**) Heatmap of representative miRNAs. RNA expression level is represented by colors, with bright blue indicating high values and bright yellow indicating low values.

RNA samples with optimal RIN (RNA Integrity Number) values (≥8) were considered to construct libraries for sequencing. Library quality was also assessed (supplementary Table S1). The libraries with sufficient concentration and a peak length within the ranges 350–500 bp and 138–150 bp were sequenced on an Illumina HiSeq platform to generate mRNA and miRNA sequences, respectively. In the present study, the mRNA sequencing generated an average of 48348613 raw reads for each sample. After quality filtering (removing adapter and low-quality reads), an average of 48309324 (99.9%) high-quality, clean reads were obtained for each sample. A large portion of clean reads (92.5%) were mapped to the rat genome. Among those reads, we observed 21174764 spliced reads and 20772406 non-spliced reads. Moreover, the miRNA sequencing yielded an average of 10088010 raw reads for each sample, and 8629406 (85.5%) clean reads were filtered out. A large number of clean reads (97.9%) were mapped to the genome. The high percentage of mapped reads is consistent with what would be expected for high-quality datasets^18^. The quality characteristics of sequence reads for each sample are listed in supplementary Table S2. Additionally, the RNA expression levels were determined by reads per kilobase per million mapped reads and are provided in supplementary Table S3. Hierarchical clustering of representative mRNA and miRNA expressions revealed samples in the same group clustered closely, which indicated high reproducibility in biological replicates (Fig. 1).

### Differentially expressed mRNAs and miRNAs

**Figure 2:**
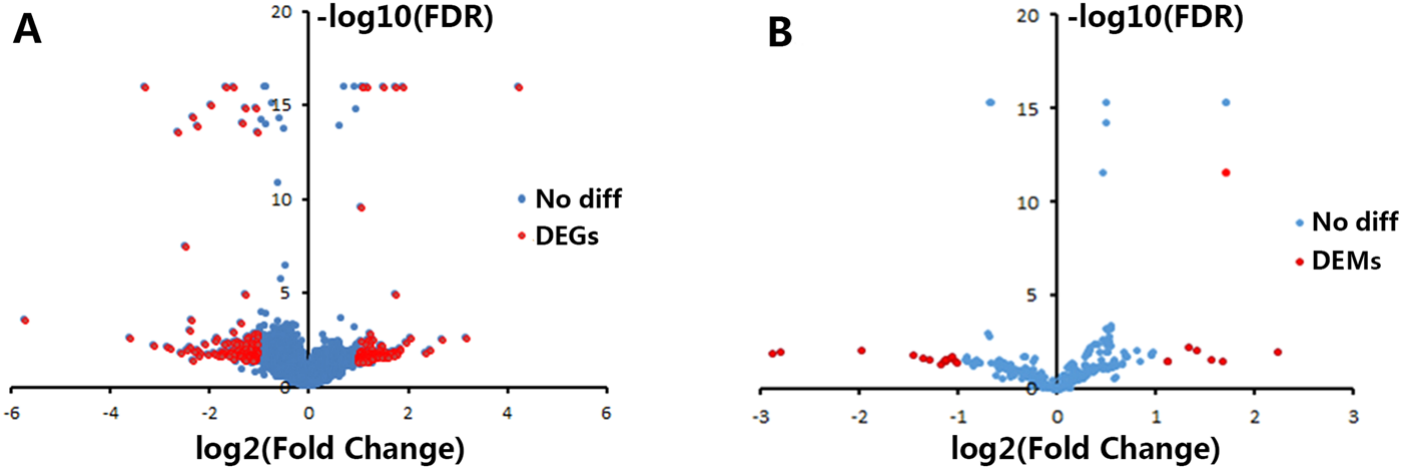
Volcano plots of mRNAs and miRNAs. The plots were constructed by plotting −log_10_ (FDR) on the y-axis and log_2_ (fold change) on the x-axis. DEGs, differentially expressed mRNAs; DEMs, differentially expressed miRNAs. (**A**) Volcano plots of mRNAs; red blots represent differentially expressed mRNAs, and blue blots represent mRNAs with no significant difference. (**B**) Volcano plots of miRNAs; red blots represent differentially expressed miRNAs, and blue blots represent miRNAs with no significant difference.

Using cutoffs of a two-fold change and an FDR less than 0.05, we identified 336 differentially expressed mRNAs (DEGs); 105 were down-regulated, and 201 were up-regulated in NAFLD rats. Among these DEGs, Abcg8, Cyp1a1, Cyp51, Hmgcr, *etc*. were decreased, whereas Apln, Cd36, Cyp7b1, Vcan, *etc*. were increased. We also identified 21differentially expressed miRNAs (DEMs). Compared with the miRNAs in the normal group, 14 were down-regulated and 7 were up-regulated in the NAFLD group. Among these DEMs, miR-33, miR-33-5p, miR-33-3p, miR-144-3p and miR-99a-3p were down-regulated in NAFLD, whereas miR-200b, miR-200b-3p, miR-200b-5p, miR-200c-3p and miR-349 were up-regulated. Volcano plots (Fig. 2) present DEGs or DEMs as red blots and other detected mRNAs and miRNAs as blue blots. Detailed information pertaining to the top 20 DEGs and total 21 DEMs is listed in Tables 1 and 2, respectively. Supplementary Table S4 lists 336 DEGs.

**Table 1:** The top 20 differentially expressed genes.

Gene_Symbol	FDR	Fold_Change
Tex13b	0.003	8.984
Cpb1	0.012	5.479
Kcnj15	0.016	5.124
Sbspon	0.003	4.145
Cxcl13	0.004	3.898
Ubd	0.000	3.744
Asns	0.012	3.580
Clec7a	0.011	3.559
Hdc	0.014	3.482
Neb	0.022	3.464
Nfe2	0.008	0.189
Tlcd2	0.011	0.185
Pcsk9	0.000	0.179
Tm7sf2	0.000	0.160
Sdr16c6	0.010	0.145
Rnf186	0.008	0.139
Igfn1	0.006	0.114
Sqle	0.000	0.102
Cish	0.003	0.082
Pnpla5	0.000	0.019

**Table 2:** The identified differentially expressed miRNAs.

miRBase	FDR	Fold_Change
rno-miR-301b-3p	0.013	0.136
rno-miR-301b	0.010	0.143
rno-miR-33-5p	0.008	0.255
rno-miR-33	0.016	0.367
rno-miR-144-3p	0.022	0.391
rno-miR-881	0.027	0.411
rno-miR-881-3p	0.046	0.446
rno-miR-219a-5p	0.031	0.453
rno-miR-3068-3p	0.031	0.459
rno-miR-99a-3p	0.026	0.460
rno-miR-33-3p	0.030	0.461
rno-miR-182	0.022	0.481
rno-miR-490	0.031	0.492
rno-miR-490-3p	0.043	0.498
rno-miR-378b	0.034	2.181
rno-miR-200c-3p	0.006	2.523
rno-miR-200b-5p	0.008	2.666
rno-miR-184	0.026	2.976
rno-miR-200b-3p	0.000	3.270
rno-miR-200b	0.000	3.296
rno-miR-349	0.011	4.750

### Annotation, target gene identification and functional enrichment

**Figure 3:**
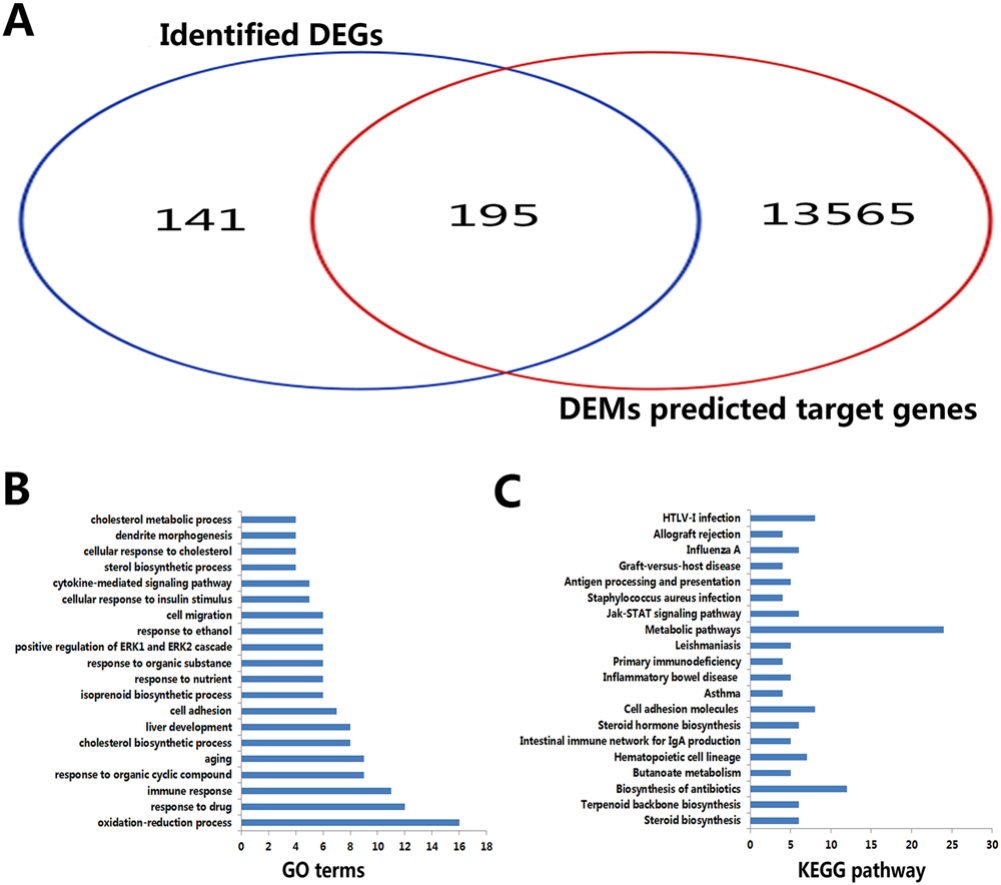
GO and KEGG pathway analysis of the 195 overlapped genes. (**A**) Venn diagram of DEM predicted target genes (13760) and identified DEGs (336) obtained from 195 overlapped genes. (**B**) The top 20 enriched GO terms; the x-axis represents gene counts, and the y-axis represents GO terms. (**C**) The top 20 enriched KEGG pathways; the x-axis represents gene counts, and the y-axis represents KEGG pathway names.

A literature search revealed that altered expression of miR-33-5p, miR-33, miR-182 and miR-200b has been reported in NAFLD, whereas the remaining 17 DEMs (e.g., miR-144-3p, miR-99a-3p, miR-200b-3p, miR-200b-5p, miR-200c-3p, miR-33-3p, miR-349, miR-184, *etc*.) have not been reported in NAFLD. Target genes of the 21 DEMs were predicted by the online tool of the miRWalk database. We obtained 13760 target genes for the 21 DEMs. A Venn diagram (Fig. 3A) showed that 195 overlapped genes were obtained between 13760 target genes and 336 DEGs, which were selected for further analysis. Detailed information pertaining to the 195 overlapped genes is provided in supplementary Table S5.

To further establish the main biological function and key pathways of the 195 overlapped genes, functional enrichment was performed by DAVID. GO analysis revealed that the overlapped genes were enriched in 47 terms such as cholesterol biosynthetic process, immune response and oxidation-reduction process (Fig. 3B). KEGG pathway enrichment showed that 20 pathways were enriched such as steroid biosynthesis, NF-κB signaling pathway and adipocytokine signaling pathway (Fig. 3C).

**Figure 4:**
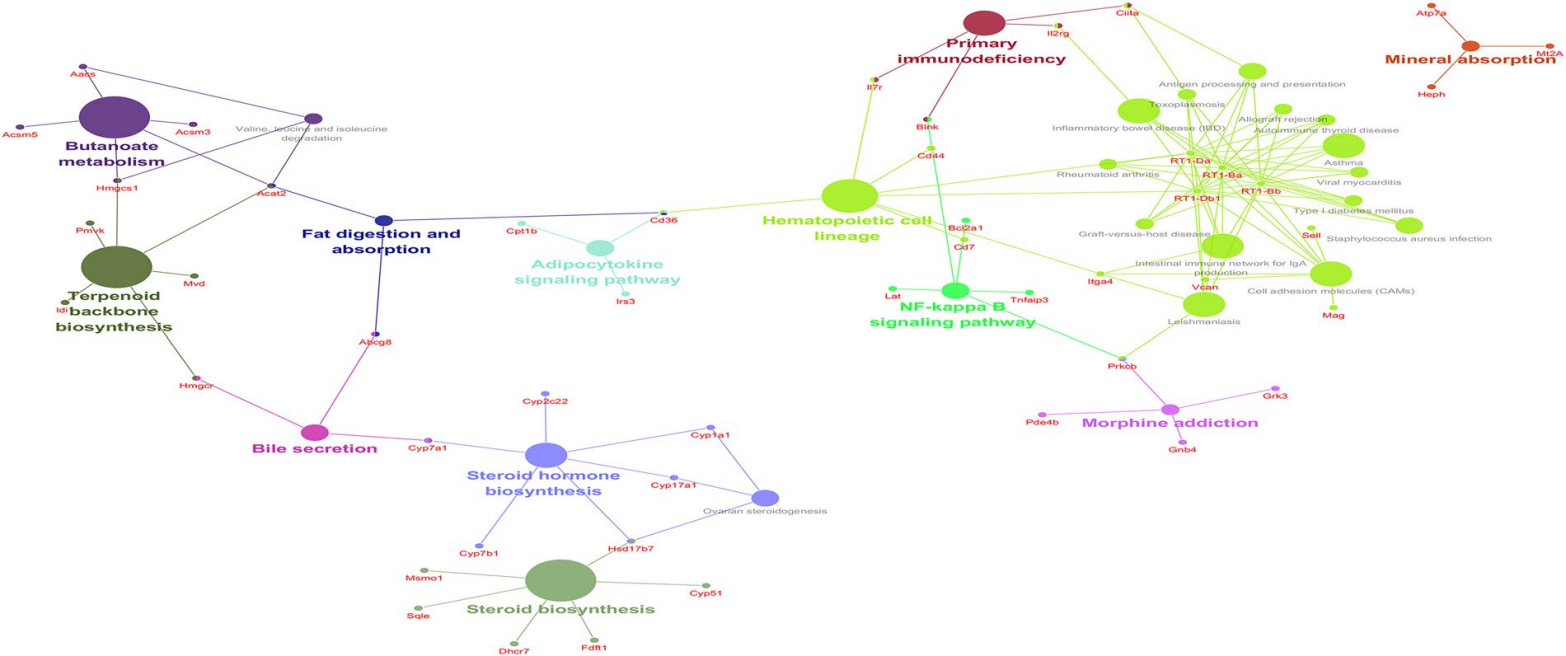
Enriched KEGG pathway interaction networks. Each node is a KEGG pathway item. The node size reflects pathway significance (FDR): the smaller the FDR is, the larger the node size is. Edge between nodes reflects common genes: the wider the edge is, the larger the overlap is. Different node colors represent different functional groups. The most significant pathway of each group features a highlighted label.

Furthermore, pathway interaction networks were constructed and are presented in Fig. 4. Pathways might interact with each other through a specific gene or hub gene. For instance, steroid hormone biosynthesis and bile secretion might interact with each other via gene Cyp7a1, while bile secretion might interact with fat digestion and absorption through gene Abcg8. RT1-Da, RT1-Ba, RT1-Bb and RT1-Db1 were hub genes of several different pathways that might interact with each other. Detailed information pertaining to genes enriched in different GO terms and KEGG pathways is provided in supplementary Table S6.

### PPI (Protein-protein interaction) network analysis

**Figure 5:**
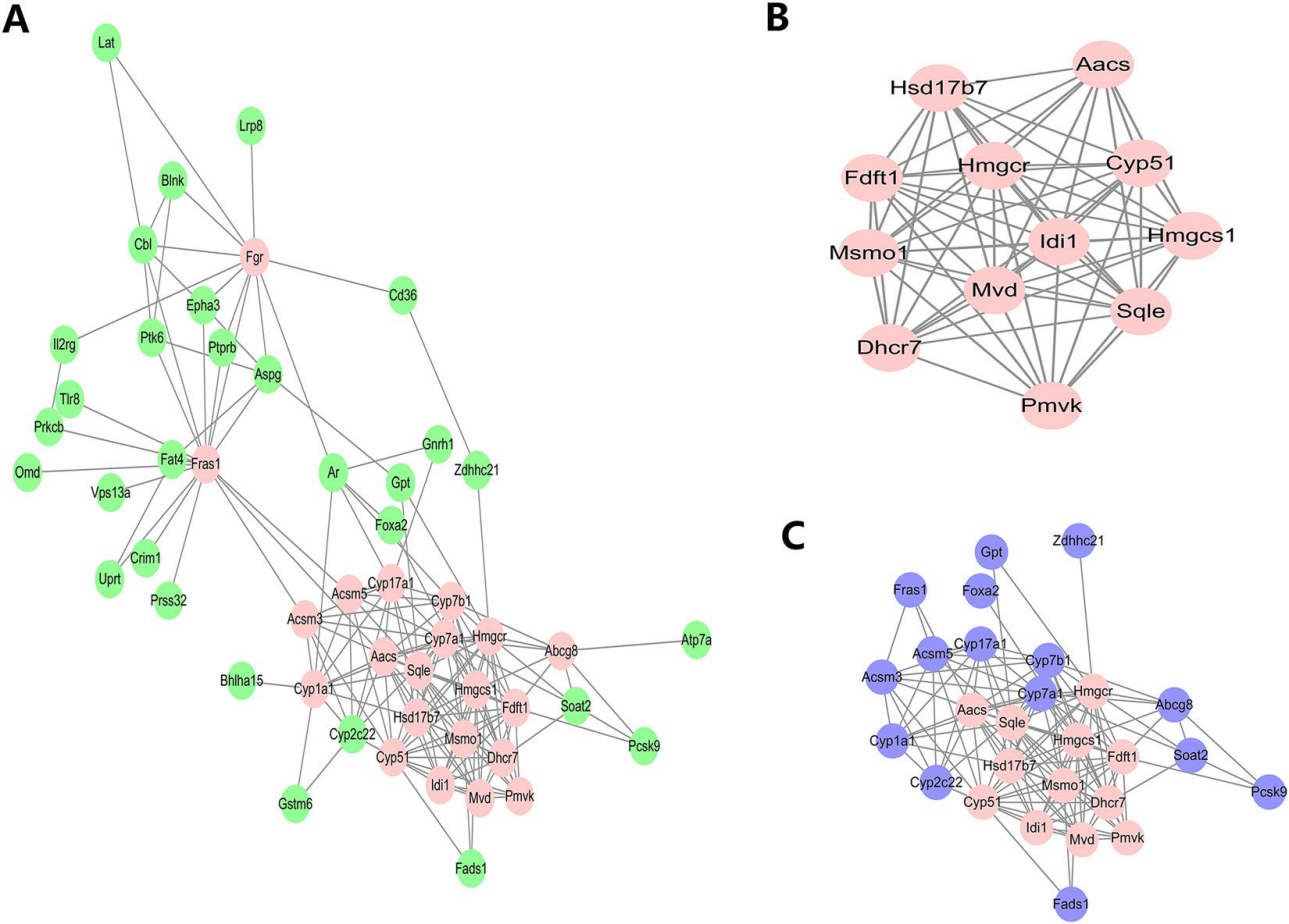
PPI networks. (**A**) PPI network with 51 nodes. Pink nodes represent the hub nodes with a large number of neighbors (≥8). (**B**) A significant module with 12 nodes identified by MCODE. (**C**) Sub-network of the most significant module with 12 nodes and their first neighbors. Blue nodes represent the first neighbors.

To explore interactions among the 195 overlapped genes, PPI was applied, and the most important modules were then screened. A PPI network with 51 nodes was obtained, and the average number of neighbors was 6.9. The hub nodes with the greatest number of neighbors (≥8), such as Abcg8, Cyp1a1, and Cyp7a1, were identified (Fig. 5A). By Molecular Complex Detection (MCODE), one significant module with the highest score (11.5) was obtained, including 12 nodes such as Cyp7b1, Cyp51, and Hmgcr (Fig. 5B). A sub-network with the first neighbors was constructed (Fig. 5C).

### Regulatory networks of differentially expressed miRNAs and mRNAs

**Figure 6:**
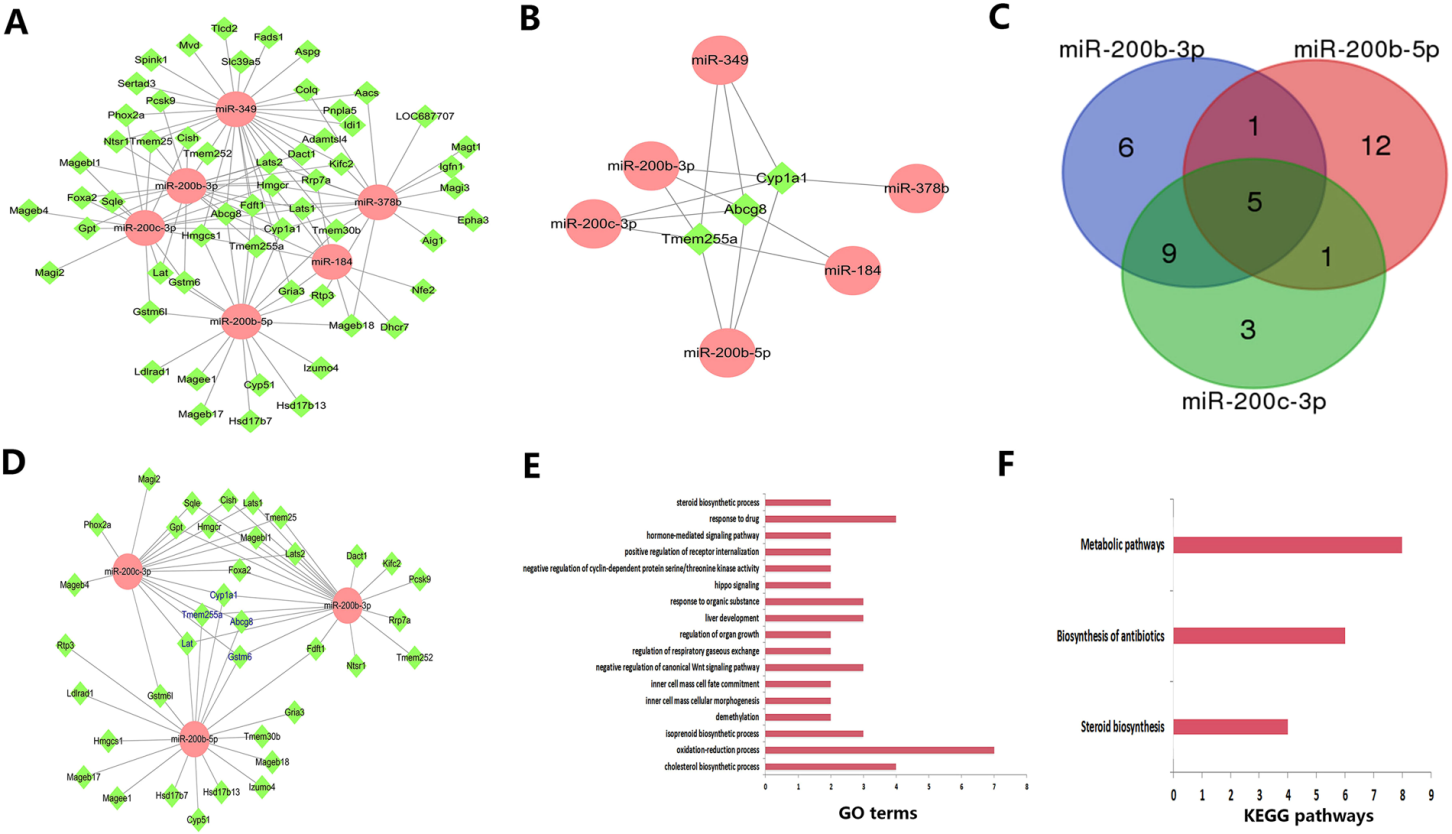
Up-regulated miRNA *vs*. down-regulated mRNA pairs. (**A**) 114 up-regulated miRNA *vs*. down-regulated mRNA pair regulatory networks with 6 miRNAs and their targets. Ellipse nodes represent miRNAs, and diamond nodes represent mRNAs; up-regulated and down-regulated miRNAs are shown in red and green, respectively. (**B**) A sub-network of Abcg8, Cyp1a1 and Tmem255a, each connected to 5 different miRNAs. (**C**) Venn diagram of miR-200b-3p, miR-200b-5p and miR-200c-3p. (**D**) A sub-network of miR-200b-3p, miR-200b-5p and miR-200c-3p and their target genes. The blue label represents the intersection target genes of the three miRNAs. (**E**) GO analysis of miR-200b-3p, miR-200b-5p and miR-200c-3p target genes; the x-axis represents gene counts, and the y-axis represents GO terms. (**F**) KEGG pathway analysis of miR-200b-3p, miR-200b-5p and miR-200c-3p target genes; the x-axis represents gene counts, and the y-axis represents KEGG pathway names.

It is well known that miRNAs play pivotal roles in regulating mRNA expression. To explore miRNA and mRNA regulatory networks in NAFLD, the expression profiles of miRNA and mRNA were combined for further analysis. Integrated analysis of 21 DEMs and 195 DEGs identified 673 negative interaction pairs. The regulatory networks were constructed and visualized by Cytoscape; 114 pairs were up-regulated miRNAs *vs*. down-regulated mRNAs (Fig. 6A), e.g., up-regulated miR-184 interacted with 9 down-regulated genes (Abcg8, Dhcr7, Hmgcr, Tmem255a, *etc*.). Notably, a single miRNA might interact with different genes, while the same gene might be a target of different miRNAs. We observed that the intersection genes Abcg8, Cyp1a1 and Tmem255a were the targets of miR-349, miR-378b, miR-200b-3p, miR-200c-3p, miR-200b-5p and miR-184 (Fig. 6B). Additionally, we observed that miR-200b-3p, miR-200b-5p and miR-200c-3p all interacted with Abcg8, Cyp1a1, Tmem255a, Gstm6 and Lat (Fig. 6C,D). GO and KEGG pathway analyses revealed that the target genes of the three miRNAs were enriched in cholesterol biosynthetic process, metabolic pathways, *etc*. (Fig. 6E,F).

**Figure 7:**
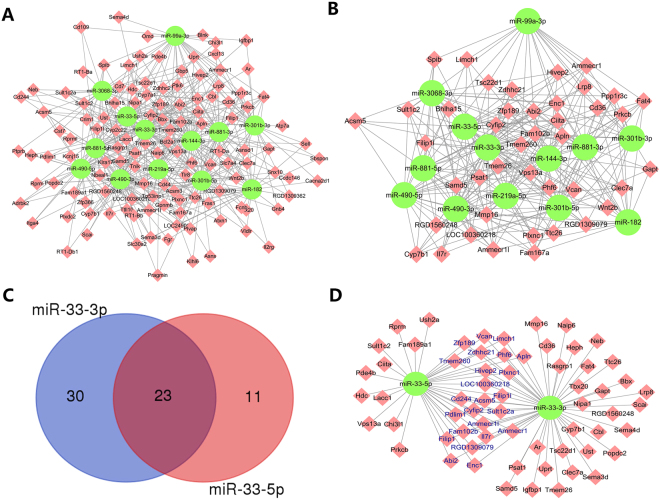
Down-regulated miRNA *vs*. up-regulated mRNAs pairs. (**A**) 459 miRNA*-*mRNA pair regulatory networks including 13 miRNAs and their targets. (**B**) A sub-network of 41 genes connected to more than 5 different miRNAs. (**C**) Venn diagram of miR-33-3p and miR-33-5p. (**D**) A sub-network of miR-33-3p and miR-33-5p and their target genes. The blue label represents the intersection target genes of the two miRNAs.

In addition, 459 pairs were down-regulated miRNAs *vs*. up-regulated mRNAs (Fig. 7A), e.g., down-regulated miR-33-5p interacted with 34 up-regulated genes (Abi2, Ciita, Sult1c2, Vcan, *etc*.). In this network, we identified a batch of genes such as Apln, Cd36, and Cyp7b1 that were connected to more than 5 miRNAs (Fig. 7B). In addition, we observed that miR-33-3p interacted with 53 mRNAs, while miR-33-5p targeted 32 genes. Furthermore, miR-33-3p and miR-33-5p targeted 23 common genes such as Abi2, Apln, and Vcan (Figs 7C,D). The miRNAs-mRNAs pairs are listed in supplementary Table S7.

### Validation by real-time PCR

**Figure 8:**
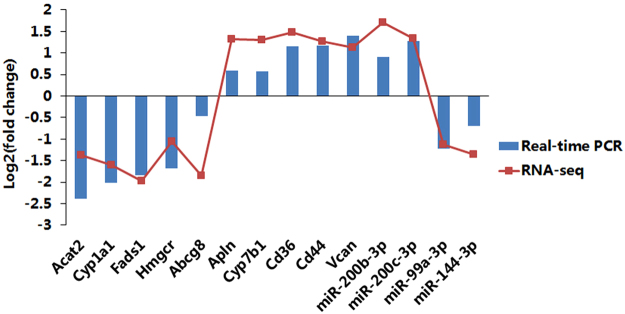
Real-time PCR validation of several representative expressed mRNAs and miRNAs. The x-axis represents RNA names, and the y-axis represents log_2_ (fold change) based on the ratios between the NAFLD and normal groups’ average expression values. Blue bars represent data yielded by real-time qPCR, and red points represent data obtained by RNA sequencing.

To validate our findings based on RNA sequence data, we selected 10 mRNAs and 4 miRNAs (half were up-regulated, and half were down-regulated) to perform real-time PCR experiments. Log_2_ (fold change) values were transformed based on the ratios between NAFLD and normal group average expression values. As shown in Fig. 8, the RNA expression patterns in real-time PCR data were consistent with RNA sequence data, which might partially validate the reliability of our sequence data and our findings in the present study.

## Discussion

In the present study, we combined the analysis of mRNA and miRNA profiles and constructed PPI, pathway interaction and miRNA-mRNA regulatory networks to enhance our understanding of molecular mechanisms of NAFLD and mine potential biomarkers.

By integrated analysis, we observed 14 decreased miRNAs (such as miR-33, miR-33-5p, miR-33-3p, miR-144-3p, and miR-99a-3p) and constructed 459 miRNA and mRNA regulatory pairs in NAFLD rats. MiR-33 is a key regulator of lipid metabolism and transport that targets ATP-binding cassette A1 (ABCA1) and thus suppresses HDL synthesis by attenuation of cholesterol efflux to apolipoprotein A1 and nascent HDL^19^. Studies have reported that genetic ablation and antagonism of miR-33 attenuates the progression of atherosclerosis in mice^20,21^. However, there are conflicting reports regarding NAFLD. A study reported that persistent inhibition of miR-33 in high-fat-diet-fed mice might cause deleterious effects such as moderate hepatic steatosis and hypertriglyceridemia^22^. Researchers observed that high-fat-diet feeding reduced miR-33 expression along with an increase in body weight, fasting blood glucose, triglyceride, cholesterol, AST and ALT in NAFLD mice^23^. Altered expression of miR-33-5p was also reported in Western-diet-induced NAFLD rats and high-fructose-diet-induced chronic metabolic disorders mice^24,25^. However, little is known about miR-33-3p in NAFLD. Intriguingly, we observed that the expression levels of miR-33, miR-33-5p and miR-33-3p were significantly decreased in high-fat-diet-induced NAFLD. All data suggested that miR-33, its guide strand miR-33-5p and passenger strand miR-33-3p might play pivotal roles in NAFLD. Moreover, the miRNA-mRNA regulatory networks revealed that 23 genes interacted with both miR-33-5p and miR-33-3p. Among those genes, Apln and Vcan have been reported to be associated with NAFLD.One study reported that the serum concentration of apelin-36 (Apln gene encoded protein) was significantly higher in NAFLD patients than in controls^26^. Another study revealed that Vcan encoded versican might serve as a novel modulator of hepatic fibrosis. Hepatic expression of versican increased in cirrhotic livers and a mouse model of fibrosis, while transient knockdown of versican inhibited expression of fibrogenic genes^27^. In the present study, we observed that Apln and Vcan were up-regulated in NAFLD, consistent with the results reported by other researchers. Collectively, there is strong evidence indicating the potential roles of miR-33-3p and its target genes in NAFLD.

Researchers reported that the serum level of miR-99a was significantly decreased in NAFLD patients compared with that in healthy controls^28^. Decreased miR-144 could enhance TNF-α and IFN-γ production, which might contribute to the progression of NAFLD in rats^29^. In addition, miR-144 might contribute to the pathogenesis of NASH in morbidly obese subjects^30^. In the present study, although we did not observe altered expression of miR-99a or miR-144, we found that miR-99a-3p and miR-144-3p were significantly down-regulated in NAFLD rats and constructed a series of miRNAs- mRNAs regulatory pairs. We also observed that NAFLD-related genes Apln, Vcan, Cd36 and Cyp7a1 were targets of miR-99a-3p and miR-144-3p^26,27,31,32^. Further investigation of the identified DEMs and their target genes may help explain the molecular mechanisms and identify novel biomarkers of NAFLD.

In addition to the down-regulated miRNAs, we obtained 7 up-regulated miRNAs (miR-200b, miR-200b-3p, miR-200b-5p, miR-200c-3p, miR-378b, miR-184, and miR-349) and 114 correlated miRNA and mRNA regulatory pairs. MiR-200b belongs to the miR-200 family, which is organized into two groups based on a single nucleotide difference in their seed sequence (group A: miR-141 and -200a; group B: miR-200b, -200c and −429)^33^. Researchers observed that expression of miR-200b and miR-200c increased in high-fat-diet-induced NAFLD rat livers and free-fatty-acid-treated HepG2 cells and human hepatocytes^34^. Another study reported that up-regulation of miR-200b in the livers of mice fed a choline- and folate-deficient diet was strongly correlated with the severity of NAFLD^35^. However, there are no reports about the correlation of miR-200b-3p, miR-200b-5p and miR-200c-3p with NAFLD. In the present study, we observed up-regulation of miR-200b in NAFLD rats, consistent with previous studies. Interestingly, we also observed that the expression levels of miR-200b-3p, miR-200b-5p and miR-200c-3p were remarkably increased in NAFLD rat livers. GO and KEGG pathway analysis revealed that their target genes were enriched in cholesterol biosynthetic process, metabolic pathways, *etc*., implying the crucial roles of the three miRNAs in NAFLD. Additionally, miRNA-mRNA regulatory network analysis indicated that five down-regulated genes (Abcg8, Cyp1a1, Tmem255a, Gstm6 and Lat) in NAFLD rats were common targets of miR-200b-3p, miR-200b-5p and miR-200c-3p. Notably, the absence of ABCG5/ABCG8 (ATP binding cassetteG5/G8) reduces biliary cholesterol secretion and results in hepatic cholesterol accumulation, acceleration of obesity, and exacerbation of NAFLD in mice^36^. It was also reported that CYP1A1 (cytochrome P450 1 A 1) protein expression was decreased in methionine-choline- deficient-diet-induced NAFLD mice and that 3,3′-diindolylmethane significantly up-regulated the protein expression of CYP1A1, which could be used as a potential therapeutic candidate for NAFLD^37^. The data presented in other studies are consistent with our findings, suggesting that miR-200b-3p, miR-200b-5p, miR-200c-3p and their target genes might be crucial regulators in the pathogenesis of NAFLD. Further investigation of the three miRNAs and their target genes may yield promising therapeutic targets of NAFLD. Additionally, miR-349 was reported to be up-regulated in hepatitis C virus-induced liver fibrosis^38^. Interestingly, we observed that miR-349 was increased in NAFLD rats, which interacted with 29 target genes, e.g., Abcg8, Cyp1a1, Fads1, Lats2, *etc*. In addition to Abcg8 and Cyp1a, Fads1 has also been reported to be correlated with NAFLD^39^. Therefore, miR-349 may serve as a new biomarker of NAFLD, which is worthy of in-depth investigation.

Furthermore, the functional enrichment of the miRNA target genes revealed that numerous genes were enriched in NAFLD-related processes and pathways such as cholesterol biosynthetic process, immune response, steroid biosynthesis, NF-κB signaling pathway, fat digestion and absorption and bile secretion^40–43^. The constructed pathway interaction networks indicated that different pathways could interact with each other through specific genes or hub genes, such as Abcg8, Acat2, Cyp7a1, Cyp1a1, Cd36, Cd44, and RT1-Ba. PPI network and MCODE module analysis also identified a batch of hub genes, including Abcg8, Cyp1a1, Cyp7b1, Cyp51, and Hmgcr. In addition to the previously mentioned Abcg8, Cd36 and Cyp1a1, genes such as Cyp7b1, Cyp51 and Hmgcr have also been reported to be correlated with NAFLD. It was reported that mRNA and protein expression levels of CYP7B1 were increased in human NAFLD^44^. Down-regulation of Cyp51 was observed by transcriptome analysis^45^. The hepatic expression of Hmgcr was reduced in morbidly obese individuals with liver steatosis and fibrosis^46^. In-depth investigation of these specific genes or hub genes may provide insight into the molecular mechanisms of NAFLD and identified novel biomarkers.

Several weak points in our present study should be noted. First, our results are based on rat models; few findings have been replicated across human studies^47^. Discrepancies in the findings are likely due to differences in study design, phenotypic endpoint, genetic alterations, *etc*. In-depth investigation should be performed to generalize our findings to humans. Second, we performed a bioinformatics analysis based on high-throughput RNA sequencing data and conducted several RNA expression validations by real-time qPCR; thorough validation and further functional experiments should be performed in the future. Third, we focused on exploring the negative miRNA-mRNA regulatory pair, considering the down-regulation role for miRNA revealed by available studies. However, recent evidence suggests that some miRNAs could activate gene expression in specific cell types and conditions with distinct transcripts and proteins. Given this complexity, further exploration should be performed to uncover the puzzling and interesting aspects of gene regulation by miRNAs.

In conclusion, our present integrated analysis may open a new avenue toward understanding the molecular mechanisms associated with the pathogenesis of NAFLD and provide potential novel biomarkers or candidate targets of NAFLD. Thorough and comprehensive investigation of the identified DEMs and their target genes should be performed in the future, which may ascertain novel biomarkers of NAFLD.

## Methods

### Animals sample collection

As previously described^17^, five-week-old male Wistar rats (130 g ± 10 g) were obtained from Shanghai SLAC Laboratory Animal CO. LTD, China, and maintained in a temperature- and humidity-controlled room. Fourteen rats were randomly divided into two groups: a normal group (n = 7), fed with a chow diet; and an NAFLD group (n = 7), fed with a high-fat diet (88% chow diet, 10% lard and 2% cholesterol)

Animals were weighed and sacrificed after 4 weeks of feeding. Liver tissues were quickly removed and stored at −80 °C after being snap frozen in liquid nitrogen. All animal procedures were approved by the Animal Experiment Ethics Committee of Shanghai University of Traditional Chinese Medicine, and all methods were performed in accordance with the relevant guidelines and regulations.

### RNA extraction and sequencing

To obtain mRNA and miRNA expression profiles, total RNA of the liver (100 mg) was extracted using TRIzol reagent (Invitrogen, USA). RNA quality was verified using an Agilent 2100 Bio-analyzer (Agilent Technologies, Santa Clara, CA). Libraries for mRNA and miRNA sequencing were respectively generated using the NEBNext Ultra RNA Library Prep Kit for Illumina (NEB,USA) and the NEBNext Multiplex Small RNA Library Prep Kit for Illumina (NEB,USA) according to the manufacturer’s instructions. Then, the final purified libraries were evaluated using a BioAnalyzer 2100 and quantified. Using the Illumina HiSeq platform, libraries for mRNA were sequenced, and 150 bp paired-end reads were generated; moreover, libraries for miRNA were sequenced, and 50 bp single-end reads were generated according to the manufacturer’s instructions. Seven biological replicate samples of each group were used, and a total of 28 libraries were sequenced.

### RNA sequencing data processing

We performed quality control on raw sequence data using the FastQC tool. To obtain high-quality, clean data for analysis, adaptor sequence trimming and removal of low-quality reads were performed^48^. Clean reads were aligned with the rat genome using TopHat v2.1.1. Based on the ultra-high throughput short-read aligner Bowtie, TopHat aligned RNA-Seq reads to reference genomes and analyzed the mapping reads to determine possible splice junctions between exons. RNA expression levels were determined by reads per kilobase per million mapped reads. Hierarchical clustering of representative mRNAs and miRNAs expressions was performed to reveal reproducibility in biological replicates.

The DESeq package was used to detect DEGs and DEMs between the normal and NAFLD groups, with thresholds of a two-fold change (the ratio between seven NAFLD rats’ and seven normal rats’ averaged signal values) and an FDR less than 0.05. The DEGs and DEMs were visualized on a volcano plot, which was constructed by plotting − log_10_ (FDR) on the y-axis and log_2_ (fold change) on the x-axis.

### Target genes prediction, functional enrichment

The target genes of DEMs were predicted by the online tool of miRWalk (http://zmf.umm.uni-heidelberg.de/apps/zmf/mirwalk2/index.html). The intersection of DEMs predicted target genes, DEGs were identified by a Venn diagram and the overlapped genes were used for further analysis.

Using DAVID 6.8 (https://david.ncifcrf.gov/), based on the default rat whole genome background, GO analysis was performed to help elucidate the concrete biological functions of specific genes, and KEGG pathways enrichment was employed to identify the critical signal pathways of the differential expressed genes. Any GO terms and KEGG pathways with p values less than 0.05 were considered significantly enriched.

### PPI, pathway interaction, miRNA-mRNA regulatory network construction

The overlapped genes were mapped to the STRING database (https://string-db.org/) to screen PPI, and networks were visualized by Cytoscape. Important hub proteins were screened by counting the degree of connectivity of each node in the network. In the network, nodes represented proteins and lines represented the interactions. The most significant modules were identified with the plug-in MCODE of Cytoscape with a cutoff MCODE score of >5. The enriched pathway interaction networks were constructed using the Cytoscape plug-ins ClueGO and CluePedia. Negatively correlated, differentially expressed miRNA-mRNA pairs were selected for screening, and the regulatory networks were constructed and visualized by Cytoscape software. Sub-networks were also constructed with mRNAs connected to a large number of different miRNAs (≥5).

### Real-time quantitative PCR assay

Total RNA was extracted from liver tissues using TRIzol (Invitrogen, China) according to the manufacturer’s protocol. For miRNA detection, total RNA was polyadenylated and then submitted to reverse transcription with primers and reverse transcriptase (Promega, USA) according to the manufacturer’s instructions. For mRNA detection, RNA was reverse transcribed into cDNA and then subjected to a real-time quantitative PCR (qPCR) assay. The qPCR assay was performed in triplicate with the SYBE Green PCR Kit on an ABI StepOnePlus Real-time PCR system (Applied Biosystems, USA). Cycle threshold (CT) values were calculated using the automated settings of the system. U6 and GAPDH were used as the internal controls for miRNAs and mRNAs, respectively. The primer sequences are listed in Tables 3 and 4.

**Table 3:** Primer information for PCR verification in miRNAs.

miRNAs	Primers sequence (5′-3′)
U6 Forward	ctcgcttcggcagcaca
U6 Reverse	aacgcttcacgaatttgcgt
miR-144-3p	ggttttttttggggtacagtatagatga
miR-99a-3p	tgggtttcaagctcgtttctatg
miR-200c-3p	gtgggtaatactgccgggta
miR-200b-3p	ttggtttttgtaatactgcctggtaat

**Table 4:** Primer information for PCR verification in mRNAs.

mRNAs	Primers
Cyp7b1	Forward tcagatgcaaagacggtcag
	Reverse cgcaggacttccatagcttc
Cd36	Forward ccagaacccagacaaccact
	Reverse cacaggctttccttctttgc
Apln	Forward gtgaagcccagaacttcgag
	Reverse atgggtcccttatgggagag
Cd44	Forward gtgtgggcagaagaagaagc
	Reverse tgttggttccttgttcacca
Vcan	Forward caccacctctgagtgggttt
	Reverse tgtgtgggctgcatttgtat
Hmgcr	Forward agaatatagcgcgtgggatg
	Reverse gacatacagccaaagcagca
Acat2	Forward caaagtggctccagaagagg
	Reverse acccatggctatggactgag
Cyp1a1	Forward ggagctgggtttgacacaat
	Reverse gatagggcagctgaggtctg
Abcg8	Forward ctacgtggacttgacgagca
	Reverse ggtgtcctgtgtgagggtct
Fads1	Forward aagcacatgccatacaacca
	Reverse cagcggcatgtaagtgaaga
GAPDH	Forward tgccactcagaagactgtgg
	Reverse ttcagctctgggatgacctt

### Statistical analysis

Data were expressed as means ± SD and were analyzed by one-way analysis of variance (ANOVA) using SPSS v18.0 software. P values less than 0.05 were considered significantly different.

### Data availability statement

All data generated during this study are included in this published article (and its Supplementary Information files).

## Electronic supplementary material

Dataset 1

Dataset 2

Dataset 3

Dataset 4

Dataset 5

Dataset 6

Dataset 7
